# Association of Meat Attachment with Intention to Reduce Meat Consumption Among Young Adults: Moderating Role of Environmental Attitude

**DOI:** 10.3390/nu17162637

**Published:** 2025-08-14

**Authors:** So-Young Kim, Min Hyun Maeng

**Affiliations:** 1 Department of Food Science and Nutrition, Soonchunhyang University, Asan 31538, Republic of Korea; 2 Department of Consumer Behavior and Family Economics, University of Wisconsin, Madison, WI 53706, USA; mmaeng@wisc.edu

**Keywords:** meat reduction, sustainable diet, plant-based diet, sustainable food choice, environment concern

## Abstract

**Background/Objectives:** Sustainability discussions are increasingly highlighting the environmental and health impacts of meat production and consumption. The study aimed to analyze how meat attachment influences the intention to reduce meat consumption among young adults, considering the moderating role of environmental attitude. **Methods:** An online survey was conducted from 27 April to 1 May 2022, among young Korean adults in their 20s, and 1478 responses were collected. The survey questionnaire assessed the participants’ (1) meat attachment (hedonism, affinity, entitlement, and dependence), (2) environmental attitude, (3) intention to reduce meat consumption, and (4) socio-demographics. Hierarchical multiple regressions were performed to explore the associations between meat attachment and the intention to reduce meat consumption, controlling for the participants’ socio-demographic characteristics. The moderating effect of environmental attitude on the associations was assessed using the PROCESS macro. **Results:** The results showed that, among the four subscales of meat attachment, affinity (β = 0.103, *p* < 0.001) and dependence (β = −0.201, *p* < 0.001) significantly predicted the intention to reduce meat consumption. In contrast, hedonism (β = −0.007, *p* = 0.831) and entitlement (β = 0.019, *p* = 0.509) exhibited no significant associations. Additionally, environmental attitude significantly moderated the associations between both affinity (interaction β = 0.135, *p* = 0.001) and dependence (interaction β = −0.159, *p* < 0.001) and the intention to reduce meat consumption. **Conclusions:** Overall, this study suggests that addressing meat dependence and affinity could be crucial for encouraging a reduction in meat consumption. Additionally, raising environmental attitude among younger adults may be needed before encouraging meat reductions. Given the limited research conducted in Asian contexts, this study offers a valuable foundation for the development of future educational interventions targeting sustainability concerns associated with meat consumption.

## 1. Introduction

Currently, discussions on sustainability increasingly focus on the production and consumption of meat and animal-sourced foods, which harm the environment in various ways [1]. Globally, over a quarter of the total GHG emissions come from the food production and consumption process, with meat and other animal-sourced foods contributing significantly more to these emissions than plant-based foods [1,2]. Red meat, in particular, accounted for over half of these emissions in 2005–2007 and is projected to continue to do so by 2050 [1]. Furthermore, animal-sourced foods generally have larger water footprints than plant-based foods, both in terms of physical weight (L/kg) and nutritional energy content (L/kcal). For example, beef’s water footprint per calorie is 20 times larger than that of cereals and starchy roots, making it the most water-intensive food product [3]. Although less water-intensive than beef, both pork and poultry still compete with human consumption for feed [3,4].

Excessive meat consumption not only has environmental implications but can also adversely affect human health. According to Godfray et al. [1], high levels of red and processed meat consumption are associated with a modest increase in total mortality rates in high-income Western countries. Wolk [5] reviewed the health risks associated with consuming 100 g of unprocessed red meat per day, ranging from non-significant to elevated risks for various major chronic diseases. Additionally, most studies in the review indicated that consuming 50 g of processed meat per day could lead to health hazards [5].

Given the significant environmental and health impacts of meat consumption, it is both necessary and effective to encourage a shift in consumer behavior to reduce it. Previous research suggests that transitioning to diets with less reliance on animal-sourced foods could mitigate the projected increase in GHG emissions caused by food production and lower risks of certain cancers and cardiovascular and chronic illnesses [2,6,7,8,9]. Adopting alternative diets such as pescatarian and vegetarian diets that incorporate fewer animal-sourced foods could lower food production emissions by 45% and 55%, respectively [3]. Gradual transitions towards plant-based diets could serve as a preventive measure against non-communicable diseases like coronary heart disease, colorectal cancer, and type 2 diabetes, supported by substantial evidence of the health benefits of these diets [10].

In 2023, global meat production reached 372.4 million tonnes [11], and this figure is expected to rise further with the economic and population growth of low- and middle-income countries, driving higher demand for meat. Most countries in Asia, Latin America, and Africa have experienced substantial increases in per capita meat consumption [12]. By 2034, the OECD and FAO project that nearly half of the global increase in meat consumption will occur in upper-middle-income countries. In contrast, high-income countries—accounting for only 17% of the global population but 35% of total meat consumption in 2024—are expected to see per capita meat intake plateau or even decline, as consumers become increasingly concerned with ethical, environmental, and health considerations [13].

Specifically, dietary and disease patterns in South Korea have dramatically changed since the 1970s, coinciding with rapid economic growth. According to Kim et al. [14], South Korea’s nutrition transition is marked by a rise in animal food product consumption and a decline in total cereal intake. Nutrient-wise, Koreans have shifted towards gaining protein from animal-sourced foods rather than from traditional sources such as rice and legumes [14]. Between 1969 and 1995, meat consumption, including poultry, surged tenfold, with the highest rate of increase occurring in the early 1980s [14]. In 2022, the per capita consumption of meat (beef, pork, and chicken) in South Korea amounted to 58.4 kg, surpassing rice (56.7 kg), a staple in Korean cuisine, for the first time [15].

The term “meat attachment” refers to a favorable connection to eating meat. Graça et al. [16] developed a four-subscale measurement of consumers’ meat attachment, representing consumers’ positive bond with meat consumption: *hedonism* (the pursuit of pleasure from consuming meat), *affinity* (the emotional connection and sense of being linked to meat consumption), *entitlement* (the belief that one has a right to consume meat), and *dependence* (the feeling of reliance on meat consumption). Studies have shown that an individual’s attachment to meat is a significant factor in predicting their willingness to reduce meat consumption and adopt a more plant-based diet [17,18,19,20,21]. Chen [22] also reported that the positive association between an individual’s attitude towards plant-based meat alternatives and their willingness to try them was moderated by their level of meat attachment.

However, previous research has typically treated meat attachment as a single, unified variable, without exploring the distinct effects of each individual subscale [17]. Moreover, majority of these studies have been conducted in Western countries, with relatively little evidence available from other regions, including Asian countries. Therefore, this study aimed to examine the association between the meat attachment among young Korean adults and their intention to reduce meat consumption, focusing on the distinctive impacts of each subscale. In addition, this study further analyzed the moderating role of environmental attitude in these associations, which has been highlighted as significant in prior studies [23,24,25,26,27]. To our knowledge, this study is one of few attempts in Asian countries to explore this topic, adding to the existing research within the broader region. The findings of this study may offer valuable insights for designing future educational interventions aimed at reducing meat consumption and promoting sustainable diets in South Korea and other countries with comparable priorities and challenges.

## 2. Methods

### 2.1. Study Design and Participants

An online survey was undertaken among individuals in their twenties in South Korea from 27 April to 1 May 2022. Prior to the main survey, a pre-testing was conducted on 22 online survey panels from 18 to 19 April 2022, to ensure the clarity, comprehensibility, and user-friendliness of the questionnaire. The website construction and participant recruitment for both the pre-testing and main surveys were managed by Macromill Embrain (www.embrain.com), a leading company specializing in online research in South Korea. Macromill Embrain is an opt-in panel provider, not a crowdsourcing platform. Individuals voluntarily register and provide demographic information upon joining, which enables the company to conduct targeted sampling based on key demographic attributes. The company maintains a panel of over 1.5 million individuals in South Korea, from which participants were recruited for this study using a non-probability, convenience sampling approach.

For data collection, self-administered questionnaires were distributed via email to members of the survey panel. To qualify for participation, individuals had to meet the following criteria: (a) being in their twenties and (b) not having dietary restrictions for health reasons. Those who met these criteria proceeded to complete the full questionnaire. To minimize potential common method bias associated with self-reported data, participant anonymity was ensured, and it was emphasized that there were no right or wrong answers. The questionnaire was carefully designed to reduce respondent fatigue and avoid priming effects by presenting items in a neutral tone and logical order. Upon questionnaire completion, participants received a token of appreciation equivalent to approximately USD 3. After excluding 105 incomplete responses, a total of 1428 questionnaires were retained for the final analysis.

This study was approved by the Institutional Review Board of Human Subjects Research and Ethics Committees of the Soonchunhyang University (Approval No. 1040875-202203-SB-034). Written informed consent was obtained from all participants prior to the survey.

### 2.2. Survey Questionnaire

The study focused on the survey results from the questionnaire related to participants’ (1) meat attachment, (2) environmental attitude, (3) intention to reduce meat consumption, and (4) socio-demographics (Appendix A). Given the specific focus of this study, additional survey results were excluded to avoid a broader and overly complex scope.

Meat attachment was assessed using the Meat Attachment Scale (MEAS), as developed by Graça et al. [19,20]. The MEAS encompasses four subscales: *hedonism* (4 items), *affinity* (4 items), *entitlement* (3 items), and *dependence* (5 items), aiming to comprehend meat consumption and potential motivations for reduction [19,20]. Initially, the MEAS was translated into Korean using a parallel back-translation procedure to ensure the preservation of the original meaning. All items were assessed using a five-point Likert scale, ranging from “strongly disagree” to “strongly agree”, with some items reverse-coded when necessary.

The environmental attitude was measured based on a review of the relevant literature (10 items) [28,29,30]. All items were measured by a five-point Likert scale, ranging from one for “strongly disagree” to five for “strongly agree.” Participants’ intention to reduce meat consumption was measured using a single Likert-type item rated on a five-point scale (1 = very unlikely to 5 = very likely). In addition, socio-demographic data including sex, education, household income, occupation, and BMI were collected.

### 2.3. Statistical Analyses

Descriptive statistical analyses were conducted for all variables in the study. The reliability of the multi-item measurement tools was assessed using Cronbach’s α, and correlations among the study variables were examined using Pearson’s correlation coefficient (r).

Subsequently, a three-step hierarchical multiple regression was executed to explore the associations between meat attachment and the intention to reduce meat consumption, controlling for socio-demographic characteristics of the participants (sex, education level, household income level, occupation, and BMI). The socio-demographic characteristics were introduced in step 1 (Model 1), the four dimensions of meat attachment in step 2 (Model 2), and environmental attitude in step 3 (Model 3).

The moderating effect of environmental attitude on the association between meat attachment and the intention to reduce meat consumption was assessed using the PROCESS macro proposed by Hayes [31]. Prior to the moderation analysis, we conducted a multicollinearity diagnostic using Variance Inflation Factors (VIFs). All VIF values were below 5.0, indicating no problematic multicollinearity among the independent variables. In addition, all continuous variables were mean-centered prior to analysis to reduce multicollinearity as per the recommendation of Aiken et al. [32]. In the event of a significant interaction, simple slopes were analyzed and depicted for meat attachment and intention to reduce meat consumption under three conditions of environmental attitude [30]: low at −1 SD, normal at the mean, and high at +1 SD.

All statistical analyses were conducted using SPSS Version 29 (IBM Corp., Armonk, NY, USA) at a significance level of 0.05.

## 3. Results

### 3.1. Participants’ Socio-Demographic Characteristics

The survey included 1478 individuals, with 47.1% being male and 52.9% female. The majority of respondents either held a college/university degree (47.8%) or were currently enrolled as college/university students (34.9%). In terms of occupation, 47.1% were employees, and 35.5% were either college/university students or graduate students. Regarding monthly average household income, 41.7% fell within the range of ≥USD 790 and <USD 2371, while 22.8% reported an income below USD 790. In terms of BMI, 52.2% of participants were classified as normal weight, and 39.3% were categorized as overweight or obese (Table 1).

### 3.2. Reliability of Multi-Item Measurements

Analyses of the reliability of the multi-item measurements for each subscale of meat attachment and environmental attitude showed Cronbach’s alpha coefficients with an excellent internal consistency of >0.7 (Table 2).

### 3.3. Associations Between Meat Attachment and Intention to Reduce Meat Consumption

Table 3 presents the results of a three-step hierarchical multiple regression analysis to assess the independent association between meat attachment and the intention to reduce meat consumption, controlling for participants’ socio-demographic characteristics. All three models (Model 1, Model 2, and Model 3) demonstrated satisfactory fits, and there were no indications of multicollinearity issues among the variables. The R-squared values for Model 1, Model 2, and Model 3 were 0.100, 0.160, and 0.217, respectively.

However, within the four subscales of meat attachment, only *affinity* (β = 0.134, *p* < 0.001) and *dependence* (β = −0.275, *p* < 0.001) emerged as significant predictors of the intention to reduce meat consumption. Conversely, *hedonism* and *entitlement* exhibited no significant associations. Notably, among socio-demographic characteristics, only the participants’ sex retained significance in the final model, demonstrating a more positive impact on the intention to reduce meat consumption for females (β = 0.421, *p* < 0.001) than for males.

### 3.4. Moderating Effect of Environmental Attitude Between Meat Attachment and Intention to Reduce Meat Consumption

Table 4 displayed the results of the PROCESS macro, investigating a moderating effect of environmental attitude on the relationship between meat attachment (*affinity*, *dependence*) and the intention to reduce meat consumption.

As shown in Table 4, the interaction between *affinity*/*dependence* and environmental attitude exhibited a significant effect with B = 0.135 (*p* < 0.001) and B = −0.159 (*p* < 0.001), respectively. The results affirm the moderating effect of environmental attitude on the relationship between *affinity*/*dependence* and the intention to reduce meat consumption.

**Figure 1:**
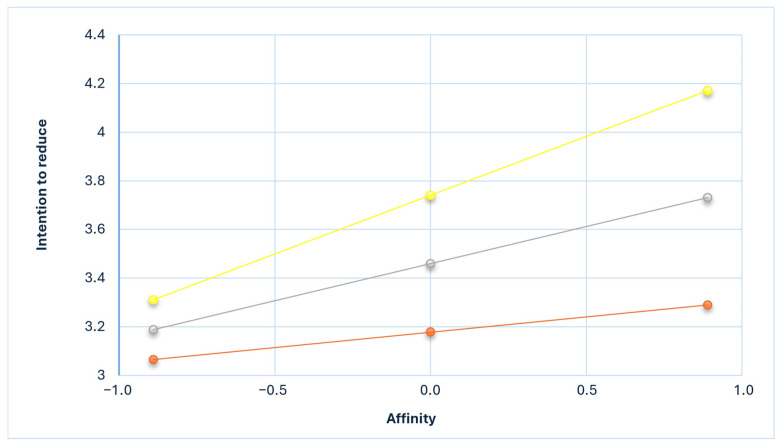
Simple slope equations of the relationship between affinity and intention to reduce meat consumption when environmental attitude is low (one SD below the mean; orange), mean (gray), or high (one SD above the mean; yellow).

Figure 1 illustrates the simple slopes between *affinity* and intention under three conditions. *Affinity* was positively associated with the intention to reduce meat consumption at low (B = 0.109, *p* < 0.01), normal (B = 0.202, *p* < 0.001), and high (B = 0.295, *p* < 0.001) levels of environmental attitude. The slope of the positive relationship between *affinity* and intention intensified as environmental attitude increased.

**Figure 2:**
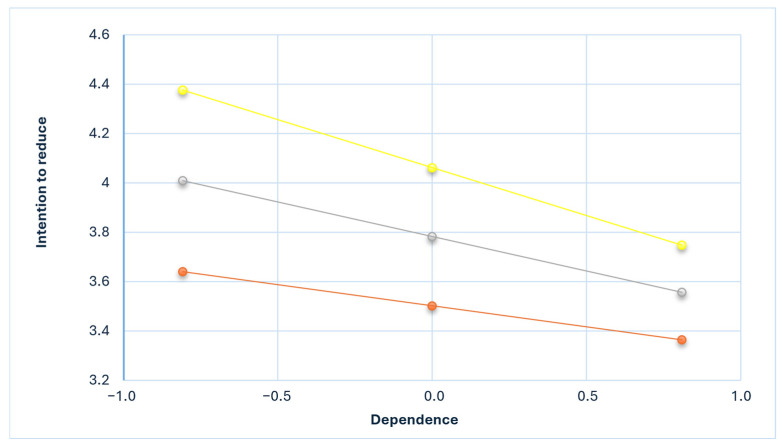
Simple slope equations of the relationship between dependence and intention to reduce meat consumption when environmental attitude is low (one SD below the mean; orange), mean (gray), or high (one SD above the mean; yellow).

Similarly, Figure 2 depicts the simple slopes between *dependence* and intention across three conditions. *Dependence* exhibited a negative association with the intention to reduce meat consumption at low (B = −0.170, *p* < 0.01), normal (B = −0.280, *p* < 0.001), and high (B = −0.389, *p* < 0.001) levels of environmental attitude. The slope of the negative relationship between *dependence* and intention attenuated as environmental attitude increased.

## 4. Discussion

This study examined the association between meat attachment and the intention to reduce meat consumption among young Korean adults in their 20s.

### 4.1. Associations Between Meat Attachment and Intention to Reduce Meat Consumption

The results revealed that, among the four subscales of meat attachment, only *affinity* and *dependence* significantly predicted participants’ intention to reduce their meat consumption.

Our results align with previous research, which underscores the crucial role of the *dependence* subscale in shaping individuals’ eating habits and decisions related to meat consumption [33,34]. The *dependence* subscale is associated with the belief that meat is essential for overall health. In other words, individuals who think their health would suffer without consuming meat are convinced that no viable replacement exists that can provide the same level of nourishment and vitality.

In a Belgian study by Roozen and Raedts [33], *dependence* was identified as the sole predictor of both meat consumption and the willingness to reduce meat intake. This study also used the 4Ns framework [35]—*normal, necessary, natural, and nice*—which are the key justifications meat eaters use to resolve the “meat paradox.” Of these, *necessary* and *normal* were significant predictors of the willingness to reduce meat intake, with *necessary* closely linked to the *dependence* subscale of meat attachment [33]. A similar outcome was observed in a Polish study by Szczebyło et al. [34], which revealed that participants following a reduced-meat diet (referred to as “testers” and “reducers”) exhibited the lowest *dependence*, highlighting this subscale as a key contrast between regular meat eaters and those with limited meat consumption.

A study by Wang and Scrimgeour [36] further explored the role of different subscales in predicting intention across cultural contexts, emphasizing *affinity, entitlement*, and *dependence* in New Zealand and *hedonism* in China. The authors attributed these differences to variations in public regulation, education, animal welfare awareness, dietary traditions, and economic development between the two countries. This cultural comparison across several Eastern and Western countries was also examined in studies by Bryant et al. [17] and Kühn et al. [37], revealing that India had the lowest attachment to meat, while the U.S. had the highest.

Additionally, the *affinity* subscale significantly predicted the intention to reduce meat consumption. Since this subscale assesses the ability to connect eating meat with the pain and death of animals, it could contribute to reducing dissociation. Dissociation is a strategy people use to disconnect meat from its animal origins to resolve cognitive dissonance; it is a significant barrier to reducing meat consumption [38,39]. Kunst and Palacios Haugestad [40] demonstrated that displaying a pork roast with its head led to reduced dissociation, increased feelings of repulsion and empathy, and ultimately a decreased intention to consume meat, with a greater likelihood of choosing a vegetarian option instead.

On the other hand, the *hedonism* and *entitlement* subscales did not predict the intention to reduce meat consumption among Korean young adults. This finding partially contrasts with previous research conducted in other countries like China [36], Poland [34], and New Zealand [21,36], where all the subscales, including *hedonism* and *entitlement*, were found to significantly predict the intention to meat reduction [21,34] or switch to plant-based diets [36]. Particularly, the Polish study emphasized *hedonism* and *entitlement*, revealing that individuals identifying as regular meat eaters rated significantly higher on statements related to *hedonism* [34]. In addition, regular meat eaters showed the highest score on a statement of *entitlement* regarding the belief in humans’ right to consume meat within the food chain hierarchy [34]. Wang and Scrimgeour [36] highlighted the relative importance of *hedonism* in China, attributing this to the high marginal utility individuals experience from meat consumption. This heightened utility stems from China’s longstanding dietary tradition of limited meat consumption, which makes the pleasure derived from eating meat particularly valuable.

### 4.2. Moderating Effect of Environmental Attitude Between Meat Attachment and Intention to Reduce Meat Consumption

Furthermore, our study found that environmental attitude plays a moderating role; as environmental attitude increases, the link between *affinity* and intention strengthens, while the link between *dependence* and intention weakens. Similarly, previous research has emphasized that prior beliefs about the negative health and environmental impacts of meat consumption can moderate the intention to reduce meat intake [23,24,26,27]. For instance, Verain et al. [27] found that only individuals already aware of sustainability issues showed changes in their dietary intentions. Vainio et al. [26] also noted that individuals with pre-existing beliefs about the adverse health and environmental effects of red meat consumption were more influenced by information promoting reduced meat consumption and plant-based alternatives, likely due to confirmation bias.

This significance of heightened environmental attitude is more evident among individuals who have already reduced or eliminated meat from their diet. Flexitarians and vegetarians, who typically have a low attachment to meat and a strong intention to reduce their consumption, tend to demonstrate high levels of environmental attitude. Van Dijk et al. [41] reported that flexitarians in Finland and the Netherlands scored significantly higher on the Food Sustainability Knowledge Questionnaire than omnivores, indicating a deeper understanding of sustainability issues related to food [42]. Based on these findings, the authors emphasized the potential for promoting plant-based diets through a better understanding of food sustainability. Krizanova et al. [43] also highlighted the benefits of encouraging individuals to prioritize environmentalism when promoting sustainable eating patterns.

### 4.3. Study Implications

Overall, this study’s results imply that addressing meat dependence could be crucial for encouraging a reduction in meat consumption. Given that the *dependence* subscale is linked to the perceived impact of diet on health, we can help reduce people’s reliance on meat by educating them about the health benefits of reducing meat consumption and adopting more plant-based diets. Kwasny et al. [24] analyzed previous research and found that educating individuals about the environmental, health, and ethical aspects of meat production and consumption is an effective strategy for encouraging a reduction in meat consumption. To enhance effectiveness, the authors recommended integrating information on both environmental and health aspects, with a particular emphasis on the adverse health impacts of meat consumption in single-framed messages [24]. This focus on health may stem from the observation that self-centered motives, such as health concerns, have a greater impact on dietary choices than altruistic motives [24,44,45]. Turnes et al. [46] corroborated this by showing that individuals were more willing to reduce meat consumption for health-related reasons. Furthermore, health concerns were more significant for flexitarians and meat reducers, whereas strict vegans and vegetarians placed greater importance on ethical considerations, such as animal welfare and environmental impact [46,47].

The *affinity* subscale also warrants attention, as it is associated with provoking emotions. Kwasny et al. [24] argue that evoking emotions such as empathy, guilt, or disgust can be more effective than merely presenting information about meat consumption and its consequences. Despite its significance to predict intentions to reduce meat consumption, the *affinity* subscale showed the lowest average scores among the four subscales of meat attachment. This low level of *affinity* observed in young Korean adults may indicate an urgent need for interventions to help reduce dissociation and increase awareness of the animal origins of the meat they consume, potentially leading to more-ethical and sustainable food choices.

Our results also highlight the need for increased efforts to raise environmental attitude among younger consumers, particularly regarding the consequences of meat production and consumption, before implementing measures to encourage them to reduce their meat intake. As mentioned earlier, individuals are generally more receptive to information about the health and environmental impacts of meat consumption when it aligns with their pre-existing beliefs. Previous research supports this confirmation bias, showing that educating individuals or providing information to enhance environmental attitude can positively influence both their intentions and actual behavior regarding meat consumption [23,24,48]. For example, Grundy et al. [23] found in their meta-review that interventions highlighting the negative environmental impacts of meat consumption consistently led to a reduction in meat consumption. Similarly, Jay et al. [48] demonstrated that a course teaching college students in the United States about the links between food choices and environmental sustainability resulted in a decreased dietary carbon footprint, as students reduced their intake of ruminant meat, while the control group showed no significant change over time.

Achieving better dietary sustainability, however, involves overcoming several challenges. One primary issue is that many individuals do not recognize the link between meat consumption and its environmental impact [21,49,50,51]. Earlier research indicates that people often perceive reducing meat consumption as less advantageous or effective than other sustainable practices. They tend to believe that actions such as purchasing food with minimal packaging, sourcing locally, and minimizing food waste are more effective in reducing the environmental impact of their food choices [21,49,51].

Moreover, there is a recognized need to improve how environmental information is presented and communicated to the public [25,52]. While informing individuals about the environmental consequences of meat production can enhance environmental attitude and intention, Sanchez-Sabate and Sabaté [25] pointed out that such information is often presented in a highly rational and detached manner. This approach may cause people to struggle in connecting their personal experiences, including their food choices, with broader environmental issues.

Supporting this, Hopwood et al. [52] found that most participants did not view meat consumption as a behavior with moral implications, even though they acknowledged the morality of environmentally sustainable actions. According to de Boer et al. [53], even environmentally conscious consumers did not respond positively to the meat–climate issue, suggesting that the connection between meat consumption and climate change might be perceived as too ambiguous and complex. This lack of clarity could lead consumers to view the environmental problem as less urgent.

In line with the authors’ recommendation, we suggest first enhancing individuals’ environmental attitude and then helping them recognize the value of reducing meat consumption. By doing so, we can enable individuals to align their behaviors with their understanding and beliefs, rather than feeling that they are compromising other values, such as enjoyment and taste. Indeed, individuals who perceived moral value in reducing meat consumption were more likely to intend to decrease their meat intake, even though most people did not view reducing meat consumption as a moral act [52].

### 4.4. Study Limitations

While the current study has shed some light on the factors influencing the intention to reduce meat consumption among young adults, particularly regarding the roles of meat attachment and environmental attitude, the findings should be interpreted with caution due to several limitations.

First, as a cross-sectional survey, it cannot establish causal relationships between variables. Second, because all data were self-reported, the findings may be subject to social desirability bias and common method bias. While participant anonymity and a carefully structured questionnaire were used to minimize such biases, the possibility remains and should be considered when interpreting the results. Third, the sample consisted exclusively of individuals in their twenties who were recruited from an opt-in online research panel. Although Macromill Embrain manages its panel based on key demographic characteristics and attempts to align it with national distributions, it does not guarantee full representativeness of the population. Therefore, caution should be exercised in generalizing the findings to the broader population of young Korean adults and other age groups. Finally, this study used a single-item measure to assess the intention to reduce meat consumption. While such measures are acceptable for clearly defined constructs and are widely used in behavioral research [54], future studies should consider using multi-item scales to enhance the reliability and validity of the measurement.

## 5. Conclusions

This study highlights how specific dimensions of meat attachment—particularly dependence and affinity—influence young adults’ intentions to reduce meat consumption, with environmental attitudes acting as a moderator. These findings suggest that integrating environmental values into health and nutrition education can enhance efforts to promote sustainable diets. In particular, university curricula and community programs targeting young adults may benefit from this approach. Situated in an Asian context, this study contributes culturally relevant insights and offers a foundation for future policy and educational strategies.

Further research should explore the gap between intention and actual behavior by incorporating longitudinal or experimental designs to assess behavioral change over time. Additionally, investigating the roles of additional psychological, social, and structural factors may offer a more comprehensive understanding of meat consumption among young adults. Cross-cultural studies could also validate the generalizability of the present findings across different Asian and non-Asian contexts.

## Figures and Tables

**Table 1:** General characteristics of participants (*n* = 1478).

Classification		*n*	%
Sex	Male	696	47.1
	Female	782	52.9
Education	High school graduation	170	11.5
	College/university students	516	34.9
	College/university graduation	706	47.8
	Graduate students or above	86	5.8
Occupation	Employed	696	47.1
	College/university or graduate students	524	35.5
	Unemployed	258	17.5
Monthly household income (USD) ^(1)^	<790 (lowest)	337	22.8
	≥790 and <2371 (low)	616	41.7
	≥2371 and <3952 (middle)	283	19.1
	≥3952 (high)	242	16.4
BMI	Underweight	125	8.5
	Normal weight	772	52.2
	Overweight/obesity	581	39.3
Total		1478	100

^(1)^ USD 1 = KRW 1265 (based on the standard exchange rate announced during the survey period).

**Table 2:** Reliability of multi-item measurements ^(1)^ (*n* = 1478).

Classification	No. of Items	Cronbach’s α	Mean	S.D.
Hedonism	4	0.891	3.56	0.94
Affinity	4	0.822	2.13	0.85
Entitlement	3	0.835	3.17	0.95
Dependence	5	0.785	3.07	0.81
Environmental attitude	10	0.804	3.84	0.69

^(1)^ The measuring items are detailed in Appendix A.

**Table 3:** Associations between meat attachment and intention to reduce meat consumption (*n* = 1478, all participants).

Variables	Model 1				Model 2				Model 3			
	b	SE	ß	t-Value	b	SE	ß	t-Value	b	SE	ß	t-Value
Constant	3.319	0.144		23.103 ***	3.319	0.144		23.103 ***	2.348	0.266		8.813 ***
Sex												
Female	0.707	0.062	0.319	11.431 ***	0.707	0.062	0.319	11.431 ***	0.421	0.062	0.190	6.754 ***
Education												
College/university students	0.074	0.119	0.032	0.623	0.074	0.119	0.032	0.623	0.042	0.112	0.018	0.372
College/university graduation	−0.022	0.094	−0.010	−0.237	−0.022	0.094	−0.010	−0.237	−0.052	0.089	−0.024	−0.590
Graduate students or above	−0.137	0.145	−0.029	−0.941	−0.137	0.145	−0.029	−0.941	−0.108	0.136	−0.023	−0.794
Monthly household income												
≥790 and <2371 (low)	−0.016	0.076	−0.007	−0.212	−0.016	0.076	−0.007	−0.212	−0.041	0.072	−0.018	−0.576
≥2371 and <3952 (middle)	0.049	0.087	0.017	0.556	0.049	0.087	0.017	0.556	0.032	0.082	0.011	0.396
≥3952 (high)	0.085	0.091	0.029	0.936	0.085	0.091	0.029	0.936	0.094	0.086	0.031	1.096
Occupation												
Employed	−0.001	0.080	−0.001	−0.017	−0.001	0.080	−0.001	−0.017	0.048	0.075	0.022	0.637
College/university or graduate students	−0.112	0.111	−0.049	−1.014	−0.112	0.111	−0.049	−1.014	−0.084	0.103	−0.036	−0.813
BMI												
Normal weight	0.113	0.103	0.051	1.097	0.113	0.103	0.051	1.097	0.122	0.096	0.055	1.269
Overweight/obesity	0.148	0.109	0.066	1.368	0.148	0.109	0.066	1.368	0.206	0.102	0.091	2.009 *
Meat attachment												
Hedonism					0.024	0.038	0.021	0.649	−0.008	0.037	−0.007	−0.214
Affinity					0.103	0.033	0.079	3.146 **	0.134	0.032	0.103	4.227 ***
Entitlement					−0.025	0.034	−0.022	−0.752	0.022	0.033	0.019	0.661
Dependence					−0.307	0.044	−0.224	−6.996 ***	−0.275	0.042	−0.201	−6.473 ***
Environmental attitude									0.420	0.041	0.262	10.318 ***
F	14.835 ***				18.515 ***				25.264 ***			
*R* ^2^	0.100				0.160				0.217			
Adjusted *R*^2^	0.093				0.151				0.208			

* *p* < 0.05, ** *p* < 0.01, *** *p* < 0.001. b = unstandardized coefficient, β = standardized coefficient, SE = standard error.” (1) Reference group (sex): male; (2) reference group (education): high school graduation; (3) reference group (monthly household income): <USD 899 (lowest); (4) reference group (occupation): unemployed; (5) reference group (BMI): underweight.

**Table 4:** Moderated regression PROCESS model predicting intention to reduce meat consumption from meat attachment (affinity/dependence) and environmental attitude (*n* = 1478).

Variables	B	SE	t	*p*	Variables	B	SE	t	*p*
Affinity	0.202	0.032	6.371	0.000	Dependence	−0.280	0.033	−8.491	0.000
Environmental attitude	0.438	0.041	10.709	0.000	Environmental attitude	0.406	0.040	10.108	0.000
Affinity × Environmental attitude	0.135	0.042	3.222	0.001	Dependence × Environmental attitude	−0.159	0.046	−3.489	0.000
F	23.986 (*p* = 0.000)					28.325 (*p* = 0.000)			
*R* ^2^	0.187					0.213			
Δ*R*^2^	0.006 (F = 10.378, *p* = 0.001)					0.007 (F = 12.175, *p* = 0.000)			

SE = standard error, t = t-statistic.

## Data Availability

The anonymous dataset is available from the authors, upon reasonable request. The data are not publicly available due to ethical restrictions, as participants provided consent for research purposes only.

## Appendix A. Survey Questionnaire

**[Meat attachment]**

***Please read each sentence carefully and indicate to what extent you agree or disagree with each statement.***

**Classification**		**Strongly** **Disagree**	**Strongly** **Agree**
Hedonism	To eat meat is one of the good pleasures in life	:___1___:___2___:___3___:___4___:___5___:	
	I love meals with meat	:___1___:___2___:___3___:___4___:___5___:	
	I’m a big fan of meat	:___1___:___2___:___3___:___4___:___5___:	
	A good steak is without comparison	:___1___:___2___:___3___:___4___:___5___:	
Affinity	By eating meat I’m reminded of the death and suffering of animals	:___1___:___2___:___3___:___4___:___5___:	
	To eat meat is disrespectful towards life and the environment	:___1___:___2___:___3___:___4___:___5___:	
	I feel bad when I think of eating meat	:___1___:___2___:___3___:___4___:___5___:	
	Meat reminds me of diseases	:___1___:___2___:___3___:___4___:___5___:	
Entitlement	To eat meat is an unquestionable right of every person	:___1___:___2___:___3___:___4___:___5___:	
	According to our position in the food chain, we have the right to eat meat	:___1___:___2___:___3___:___4___:___5___:	
	Eating meat is a natural and undisputable practice	:___1___:___2___:___3___:___4___:___5___:	
Dependence	I don’t picture myself without eating meat regularly	:___1___:___2___:___3___:___4___:___5___:	
	If I couldn’t eat meat I would feel weak	:___1___:___2___:___3___:___4___:___5___:	
	I would feel fine with a meatless diet	:___1___:___2___:___3___:___4___:___5___:	
	If I was forced to stop eating meat I would feel sad	:___1___:___2___:___3___:___4___:___5___:	
	If I was forced to stop eating meat I would feel sad	:___1___:___2___:___3___:___4___:___5___:	

**[Environmental Attitude]**

***Please read each sentence carefully and indicate to what extent you agree or disagree with each statement.***

**Items**	**Strongly** **Disagree**	**Strongly** **Agree**
The environmental crisis facing humanity is very serious.	:___1___:___2___:___3___:___4___:___5___:	
Plants and animals are equally precious beings, just like people.	:___1___:___2___:___3___:___4___:___5___:	
If the destruction of nature continues as it is now, we will soon experience a serious environmental disaster.	:___1___:___2___:___3___:___4___:___5___:	
Environmental pollution is becoming more serious, but there is no need to worry because nature has an excellent ability to purify itself.	:___1___:___2___:___3___:___4___:___5___:	
Nature is very resilient and can be utilized to suit human needs.	:___1___:___2___:___3___:___4___:___5___:	
I feel guilty when I do something harmful to the environment.	:___1___:___2___:___3___:___4___:___5___:	
I do not believe that my daily actions and lifestyle are causing climate crisis.	:___1___:___2___:___3___:___4___:___5___:	
I get angry when I see the environment being polluted due to our careless behaviors.	:___1___:___2___:___3___:___4___:___5___:	
I am willing to share information about environmental issues with my family and friends.	:___1___:___2___:___3___:___4___:___5___:	
Even if it is not immediately effective, personal sacrifice and volunteer to reduce environmental pollution are necessary.	:___1___:___2___:___3___:___4___:___5___:	

**[Intention]**

***Please read each sentence carefully and indicate to what extent you agree or disagree with each statement***

Do you have an intention to reduce meat consumption?	Very unlikely	Very likely
	:___1___:___2___:___3___:___4___:___5___:	

**[Socio-demographic Characteristics]**

***Please answer each of the following questions.***

Please indicate your sex	① Male ② Female
Please write down your age	Age:
Please indicate the highest level of education you have completed	① High school graduation ② College/university students ③ College/university graduation ④ Graduate students ⑤ Post-graduate degree
Please indicate the level of average monthly household income (KRW)	① <1,000,000 ② ≥1,000,000 and <2,000,000 ③ ≥2,000,000 and <3,000,000 ④ ≥3,000,000 and <4,000,000 ⑤ ≥4,000,000 and <5,000,000 ⑥ ≥5,000,000 and <6,000,000 ⑦ ≥6,000,000 and <7,000,000 ⑧ ≥7,000,000
What is your current employment status?	① Self-employed ② Professionals ③ Office workers ④ College/university students ⑤ Graduate students ⑥ Short-term temporary worker (part-time worker, intern) ⑦ Unemployed ⑧ Others ( )
What is your Height and Weight?	________cm ________kg
